# Event and Comment

**Published:** 1917-06

**Authors:** 


### EVENT AND COMMENT

DEDICATION ROCHESTER DISPENSARY. An event of unusual significance was the dedication, May 9, 1917, of the new building for the Rochester, N. Y., Dental Dispensary. This building is the outcome of an effort by the dentists of Rochester to do charity work for the needy school children in that city begun several years ago by voluntary effort, but more recently carried on by gifts by interested citizens and appropriations by the city authorities. Two years ago Mr. George Eastman, of Rochester, agreed to put up a suitable building for a permanent clinic, and endow it amply for the most complete service possible. His gifts have been amply supplemented by gifts of other citizens of Rochester, and the school board of the city of Rochester, so that it will have ample funds to carry on a great work for relief and preventive dentistry.

The building is ample in size and beautifully designed, and is admirably arranged and equipped for every conceivable branch of service of a dental character. A large clinic room, equipped with specially designed chairs, cabinets, engines and other accessories, the gift of the Ritter Estate, is certainly admirably adapted for the purposes, and should contribute greatly to the comfort and efficiency of the work to be undertaken. Another department of far-reaching importance is set apart for the orthodontic treatments. The space is ample and admirably arranged. X-ray and photographic rooms of the most modern and complete character are associated with this department. A complete surgery and hospital with up-to-the-minute equipment is one of the most interesting, and, we predict, most useful, as well as history making departments in the building. The intention is to specialize in the treatment of cleft palate operations and other deformities of children. Those who know of the wonderfully successful results in relieving these dreadful deformities in little babes, by Brophy and others, will appreciate the motives which induced the management to supply a suitable place for these operations. Nothing so adequate has been so far provided in any other institution, and we shall watch its career with the greatest interest.

Another interesting feature is a research laboratory, which is to be equipped by the widow of Dr. Hoffheinz as a memorial to his great love and devotion to the profession of dentistry. The building has a beautiful room set apart for a library and museum, and another for a lecture hall. Ample rooms are also set apart for the school which has been started for the education of dental hygienists, which is destined to become a very important part of the dispensary. The first class of dental hygienists, about fifty in number, will be graduated this month. They have had eight months of study and practical training under the direction of special graduate dental instructors. The course of instruction has been given in the Rochester University building, but will hereafter be given in the Dental Dispensary.

With such facilities and so cordially supported by the loyal profession of Rochester, and with such high ideals and opportunities, we are wondering if this may not be ''the next great step in Preventive Medicine,'' which Dr. Charles Mayo suggested that dentistry would take. If it does not prove to be a great step in preventive dentistry there will need to be some gross and inexcusable blundering on someone's part. It seems that this building and its ideals have been so carefully worked out that we are destined to see many other similar institutions when the economic values in service to humanity are materialized and demonstrated. The work of such an institution, because of its permanence and scientific management, will demonstrate conclusively the importance of a carefully designed and persistent effort, rather than the unorganized and volunteer movements with their many shortcomings, which have characterized our benevolent efforts heretofore.

THE PROFESSION AND WAR. That the dental profession is getting ready to do its part in the great world war, in which we are now to be an important factor, is evident from the activities noticed in all directions. Students just completing their college courses are offering their services by the hundreds and many practitioners are planning to give up their practices and join in the humane service at the front. In fact, those who can not engage in the work actively are planning to contribute substantially to the great effort in every possible way. At first there seemed to be considerable misdirected planning and unnecessary excitement, but we now believe a systematic and orderly procedure is being put into effect that must develop an effective professional contribution of real value. The most promising effort we know of is the suggestion of the Dentists' Preparedness League that the colleges give a course of instruction in dental military preparation to the senior class and such dental practitioners as may have in mind to enter the service in the capacity of oral surgeon. Several schools are giving such a course during the last two weeks of the course. For general information we print below the schedule as given at the University of Michigan:

Course for war dental surgery, given by the College of Dental Surgery, University of Michigan, May 28 to June 9, 1917.

Three lectures will be given each day and the afternoons will be devoted to clinics and military drills.

Monday, May 28, 9 a. m. Enrollment and announcements, Dr. Ward. 10 a. m. Opening session, Dr. Darling. 11 a. m. Anatomy of head and face, Dr. Lyons.

Tuesday, May 29, 8 a. m. Hospital practice, Dr. Darling. 9 a. m. Anatomy of head and face, Dr. Lyons. 10 a. m. Oral sepsis, Dr. Bunting.

Wednesday, May 30, 8 a. m. General anesthesia, Dr. Darling. 9 a. m. Conductive anesthesia, Dr. Lyons. 10 a. m. Nitrous oxide, Dr. Loeffler.

Thursday, May 31, 8 a. m. Oral prosthesis, Dr. Hoff. 9 a. m. Conductive anesthesia, Dr. Lyons. 10 a. m. Military science, Col. Pack.

Friday, June 1, 8 a. m. Gun shot wounds, Dr. Darling. 9 a. m. Oral prosthesis, Dr. Whitman. 10 a. m. Military science, Col. Pack.

Saturday, June 2, 8 a. m. Gun shot wounds, Dr. Darling. 9 a. m. Oral sepsis, Dr. Bunting. 10 a. m. Military law, Dean Bates.

Monday, June 4, 8 a. m. Gun shot wounds, Dr. Darling. 9 a. m. War dental surgery, Dr. Lyons. 10 a. m. Filling material, Dr. Ward.

Tuesday, June 5, 8 a. m. X-ray, Dr. Loeffler. 9 a. m. War dental surgery, Dr. Lyons. 10 a. m. Diseases of army life, Dr. Rickert.

Wednesday, June 6, 8 a. m. Plastic surgery, Dr. Darling. 9 a. m. War dental surgery, Dr. Lyons. 10 a. m. First aid, Dr. Malejan. 11 a. m. Dental surgery at the front, Dr. Meyers.

Thursday, June 7, 8 a. m. Oral prosthesis, Dr. Hoff. 9 a. m. War dental surgery, Dr. Lyons. 10 a. m. Bandaging and splinting, Dr. Malejan. 11 a. m. War dental surgery, Dr. Meyers.

Friday, June 8, 8 a. m. Plastic surgery, Dr. Darling. 9 a. m. War dental surgery, Dr. Lyons. 10 a. m. Tetanus, Dr. Rickert. 11 a. m. Army dental service, Dr. Boak.

Saturday, June 9, 8 a. m. Bone grafting, Dr. Darling. 9 a. m. War dental surgery, Dr. Lyons, 10 a. m. Army dental service. Dr. Boak.

Over fifty dental practitioners took this course, besides all the senior students.

